# The impact of pulmonary metastasectomy in patients with previously resected colorectal cancer liver metastases

**DOI:** 10.1371/journal.pone.0173933

**Published:** 2017-03-22

**Authors:** Armin Wiegering, Johannes Riegel, Johanna Wagner, Volker Kunzmann, Johannes Baur, Thorsten Walles, Ulrich Dietz, Stefan Loeb, Christoph-Thomas Germer, Ulrich Steger, Ingo Klein

**Affiliations:** 1 Department of General, Visceral, Vascular and Pediatric Surgery, University Hospital, University of Wuerzburg, Oberduerrbacherstr. Wuerzburg, Germany; 2 Department of Biochemistry and Molecular Biology, University of Wuerzburg, Oberduerrbacherstr. Wuerzburg, Germany; 3 Department of Internal Medicine II, University of Wuerzburg Medical Center, Oberduerrbacherstr. Wuerzburg, Germany; 4 Comprehensive Cancer Centre Mainfranken, University of Wuerzburg Medical Center, Josef-Schneiderstr. Wuerzburg, Germany; 5 Department of Cardiothoracic Surgery, University of Wuerzburg Medical Center, Oberduerrbacherstr. Wuerzburg, Germany; 6 Mathias-Spital Rheine, Frankenburgerstr. Rheine; Germany; National Cancer Center, JAPAN

## Abstract

**Background:**

40–50% of patients with colorectal cancer (CRC) will develop liver metastases (CRLM) during the course of the disease. One third of these patients will additionally develop pulmonary metastases.

**Methods:**

137 consecutive patients with CRLM, were analyzed regarding survival data, clinical, histological data and treatment. Results were stratified according to the occurrence of pulmonary metastases and metastases resection.

**Results:**

39% of all patients with liver resection due to CRLM developed additional lung metastases. 44% of these patients underwent subsequent pulmonary resection. Patients undergoing pulmonary metastasectomy showed a significantly better five-year survival compared to patients not qualified for curative resection (5-year survival 71.2% vs. 28.0%; p = 0.001). Interestingly, the 5-year survival of these patients was even superior to all patients with CRLM, who did not develop pulmonary metastases (77.5% vs. 63.5%; p = 0.015). Patients, whose pulmonary metastases were not resected, were more likely to redevelop liver metastases (50.0% vs 78.6%; p = 0.034). However, the rate of distant metastases did not differ between both groups (54.5 vs.53.6; p = 0.945).

**Conclusion:**

The occurrence of colorectal lung metastases after curative liver resection does not impact patient survival if pulmonary metastasectomy is feasible. Those patients clearly benefit from repeated resections of the liver and the lung metastases.

## Introduction

Colorectal carcinoma (CRC) is the most common cancer of the gastrointestinal tract and the second most common cause of cancer-related deaths both in the United States and Europe [1]. About half of all patients develop distant metastases, either as synchronous metastases diagnosed at the time of initial detection of cancer or in the follow-up period as metachronous metastases [2]. These distant metastases are mainly located in the liver (CRLM). Over the past two decades, resection of CRLM has increased significantly and has led to a long-term survival of up to 50% after curative liver resection [3]. The second most common organ, in which distant metastases arise, is the lung and around 10% of the patients with CRC will develop pulmonary metastases [4,5]. The five-year survival rate of these patients without surgery is assumed to be below 5%. Similar to liver metastases, the resection of pulmonary metastases has increased during the last decade, leading to five-year survival rates of up to 68% for patients after metastases resection [6–10].

The introduction of multimodal treatment options, including chemotherapy and surgery, has resulted in a dramatic survival benefit for patients with metastatic disease. After curative metastases resection patients benefit from the surgery with an increased survival rate, which has led to surgery being introduced as the gold standard in this selected patient population.

Around 10–20% of patients with CRC will develop both liver and lung metastases. So far, the benefit of surgical resection of pulmonary metastases, arising either simultaneously or after the resection of liver metastases, is discussed controversially in the literature. Similar to liver metastases, several factors have been identified as being associated with negative outcome, such as short disease-free survival, high carcinoma embryonic antigen (CEA), as well as the number and size of metastases [10–12]. Moreover, several studies focused on the outcome after pulmonary metastasectomy and found prior liver resection to be a negative predictive marker [13,14].

The number of patients with metastatic colorectal disease being treated with a multimodal therapy approach is rapidly increasing. Therefore, it is of great interest to further stratify treatment options for a subgroup of patients presenting with pulmonary metastases, either synchronous or metachronous with regard to CRLM. The aim of this study was to evaluate the oncological outcome after pulmonary metastasectomy in patients with previous liver resection for CRC metastases.

## Patients and methods

### Patient population

All patients with colorectal liver metastases treated at the University of Wuerzburg Medical Centre (UKW) between January 2003 and May 2013 were registered in the Wuerzburg Institutional Database (WID).

### Data source

The WID is a central data repository, which has been continuously expanded on a daily basis since 1984 with clinical, operative and research data of patients, who were evaluated and treated at the UKW. The collection of data and scientific analysis was approved by the institutional review board (“Ethik-Komission bei der Medizinischen Fakultät” #2017011001). The UKW is one of three institutions in an area with a population of about 515,000 to treat patients with CRC. Data available within the WID include patient demographics, histological diagnoses based on coding standards of the International Classification of Diseases, physician data, inpatient admission and outpatient registration data, operative procedures, laboratory results and computerized pharmacy records. Continuous cross platform integration with the Wuerzburg Comprehensive Cancer Registry ensures updated follow-up information for identification of deceased patients. Inpatient and outpatient records of all identified patients were reviewed retrospectively to extract information regarding type and duration of chemotherapy, sites of metastatic disease at presentation and disease status at last follow-up. Missing data was retrieved from patient case notes when possible.

Demographic details were compiled, along with clinical variables recorded at the time of primary diagnosis as well as during the initial operation (tumor site and the presence of any metastases) and histological details of the resected specimen (tumor (T) stage, nodal (N) stage, tumor differentiation (G) and evidence of microscopic venous (V) and lymphatic vessel invasion (L)). This data was correlated with survival data obtained from prospective follow-up.

### Follow-up

Postoperative follow-up consisted of quarterly outpatient assessments or the gathering of complete information from patients’ primary care physician in 3-month intervals for at least 10 years. After 10 years, information was gathered retrospectively on an annual basis. Follow-up was performed by protocols according to entity and tumor stage with abdominal ultrasound after 3, 6, 12 and 18 months and after that on a yearly basis. Computer tomography and surveillance colonoscopy were performed routinely 3 or 6 months after the operation and were repeated every year. After 5 years, structured follow-up ceased and diagnostic tests were based on symptoms or incidental findings and initiated according to individual cases.

### Statistical analysis

The data was analyzed with a statistical software set up in Linux by an in-house biostatistician. Clinical and histological parameters were compared with the Mann–Whitney U or Kruskal–Wallis test for continuous data and with the χ2 test for categorical variables. P<0.05 was considered statistically significant. Cox proportional hazard modeling or ‘Cox regression’ was used for multivariate testing. Survival curves were drawn according to Kaplan–Meier methods.

### Ethic statement

The study was performed with permission of the local ethics committee (#2017011001). The head of the board for internal data requests, Dr. U Maeder granted permission to access data from the registry. All patients provide informed written consent to have their medical record data used in research.

## Results

Between January 2003 and May 2013, 137 patients underwent curative resection of liver metastases at the University Hospital of Wuerzburg. The median age was 64.1 years (SD 11.05, range 27.67–84.54), 70.1% (96 male, 41 female) were male. Of these patients, 53 were diagnosed with pulmonary metastases in addition to CRLM, 8 with synchronous, 45 with metachronous metastases in relation to the diagnostic point in time of liver metastases.

Patients with additional pulmonary metastases did not differ in age, sex, performance status, location and classification of the primary cancer (T-stage, N-stage and UICC-stage), as well as the time of liver metastasis occurrence (synchronous / metachronous) from those patients without pulmonary metastases. However, primary tumors of patients with additional pulmonary metastases showed less venous infiltration in the pathological staging (summary of data in Table 1).

**Table 1:** Clinical and demographic characteristics of 137 patients undergo liver resection at the university hospital Wuerzburg according to additional pulmonary metastases.

Characteristic		Liver and lung metastases (n = 53)		Only liver metastases (n = 84)		p-value
		No.	%	No.	%	
Sex	Male	33	62.3	63	75.0	0.113
	Female	20	37.7	21	25.0	
Age	Mean (SD)	62.59 (10.01)		64.99 (11.62)		0.216
	Range	38.03–76.09		27.67–84.54		
BMI in kg/m^2^ (SD)		26.57 (SD 4.31)		26.80 (SD 4.19)		0.761
Primary tumor location	Colon	32	60.4	60	71.4	0.180
	Rectum	21	39.6	24	28.6	
Primary UICC-Stage	I	1	1.9	1	1.2	0.871
	II	9	17.0	17	20.5	
	III	17	32.1	22	26.5	
	IV	26	49.1	43	51.8	
	unknown	-	-	1		
primary T-Stage	pT0	0	0.0	1	1.2	0.695
	pT1	2	3.8	2	2.4	
	pT2	5	9.4	8	9.6	
	pT3	35	66.0	61	73.5	
	pT4	11	20.8	11	13.3	
	unknown	-	-	1	-	
Primary N-Stage	pN0	18	34.0	31	37.3	0.922
	pN1	17	32.1	25	30.1	
	pN2	18	34.0	27	32.5	
	unknown	-	-	1	-	
Primary Grading	1	3	6.1	0	0.0	0.119
	2	37	75.5	67	82.7	
	2–3	0	0.0	1	1.2	
	3	9	18.4	13	16.0	
	unknown	4	-	3	-	
Primary L-STAGE	0	26	63.4	26	45.6	0.082
	1	15	36.6	31	54.4	
	unknown	12	-	27	-	
Primary V-STAGE	0	35	85.4	37	66.1	0.039
	1	6	14.6	13	23.2	
	2	0	0.0	6	10.7	
	unknown	12	-	28	-	
KARNOFSKY-INDEX	70	0	0.0	1	3.6	0.443
	80	3	15.8	3	10.7	
	90	4	21.1	11	39.3	
	100	12	63.2	13	46.4	
	unknown	34	-	56	-	
Type of liver metastasis	Synchronous	25	47.2	47	56.6	0.281
	Metachronous	28	52.8	36	43.4	

Of the 53 patients with additional pulmonary metastases, 22 (41.5%) underwent curative resection, in three (5.7%) patients a partial, most likely non curative, resection was performed and 28 (52.8%) did not undergo surgery for their pulmonary metastases for various reasons. Among these twenty-eight patients, three patients showed a diffuse lung metastatic pattern not suitable for resection, nine presented a recurrence of their liver and pulmonary metastases, eleven patients had additional metastases other than in the lung or liver, in two cases a multidisciplinary watch and wait decision was made, one patient showed a complete response following chemotherapy, and in two cases the reason for not undergoing surgery was unknown. The above mentioned three patients with partial, most likely non curative, resection were excluded from further analysis. The decision for pulmonary resection was made in a multidisciplinary team round according to operation technique and oncological reasons.

No differences in main demographic and clinical parameters were detected when comparing the patients, who underwent resection, with those, who did not undergo resection of pulmonary metastases (Table 2). When comparing the pathological analysis of resected metastases to the radiological analysis of metastases in the non-resected group, there was a trend to a higher number of metastases in the group without resection compared to the resected group, though not reaching statistical significance. There was also a trend to lower CEA-levels in the pulmonary resection group, also not reaching statistical significance (10.7μg/l vs. 92.4μg/l; p = 0.06).

**Table 2:** Characteristics of patients with pulmonary metastases after liver metastasectomy according to treatment.

Characteristic		Lung metastases resected (n = 22)		Lung metastases not resected (n = 28)		p-value
		No.	%	No.	%	
Sex	Male	12	54.5	20	71.4	0.217
	Female	10	45.5	8	28.6	
Age	Mean (SD)	62.42 (8.39)		63.33 (11.54)		0.756
	Range	42.19–73.60		38.03–76.09		
Primary tumor location	Colon	11	50.0	18	64.3	0.31
	Rectum	11	50.0	10	35.7	
Primary UICC-Stage	I	0	0.0	1	3.6	0.294
	II	5	22.7	3	10.6	
	III	5	22.7	12	42.9	
	IV	12	54.5	12	42.9	
Type of liver metastases	Synchronous	11	50.0	12	42.9	0.615
	Metachronous	11	50.0	16	57.1	
Recurrence liver metastasis	yes	11	50.0	22	78.6	0.034
	no	11	50.0	6	21.4	
Time between liver resection and occurrence of pulmonary metastases in days (unknown = 1)	Mean (SD)	147.64 (387.77)		578.19 (655.66)		0.009
	Range	(-798)–916		64–2646		
Number of pulmonary metastase	1	8	36.4	7	25.0	0.120
	2–5	9	40.9	4	14.3	
	>6	5	22.7	11	39.3	
	unknown	-	-	6	21.4	
CEA-Level at detection of pulmonary metastasis in μg/l		10,74 (+/- 30.75		92.42 (+/- 185.5)		0.06

The median follow-up for all patients was 37.97 months, with a median survival of 76.78 months. The median time span from liver resection to the occurrence of pulmonary metastasis was 288.5 days (range: -798 to 2646 days). The time span was shorter for patients, who had pulmonary resection, than for those, who did not (147 days vs. 578 days; p = 0.009). This result was greatly influenced by three patients, who underwent pulmonary metastasectomy prior to liver resection in synchronous liver and lung metastases. When only analyzing the metachronous metastasis there is no significant difference between these two groups (362 days vs. 578 days; p = n.s.).

Compared to patients without pulmonary metastases, those with additional pulmonary metastases developed a recurrence of their liver metastasis and other extra-pulmonary metastases significantly more often (66.0% vs. 34.5%; p <0.001 and 54.7% vs 21.4%; p<0.001). This reflects a more advanced stage of the disease.

Analyzing the group of patients with additional pulmonary metastases following result was found: those, who underwent resection, showed a less likely recurrence of their liver metastases compared to those, who did not undergo surgery (50% vs. 78.6%, p = 0.034). However, the percentage of patients with a recurrence of extra-hepatic metastases did not differ in these two patient groups (54.5% vs. 53.6%, p = 0.945) (Table 3).

**Table 3:** Rate of recurrence for patients with pulmonary metastases.

Characteristic		Lung metastases resected (n = 22)		Lung metastases not resected (n = 28)		p-value
		No.	%	No.	%	
Recurrence of liver metastases	yes	11	50.0	22	78.6	0.034
	no	11	50.0	6	21.4	
Extrahepatic recurrence	yes	12	54.5	15	53.6	0.945
	no	10	45.5	13	46.4	

**Fig 1:**
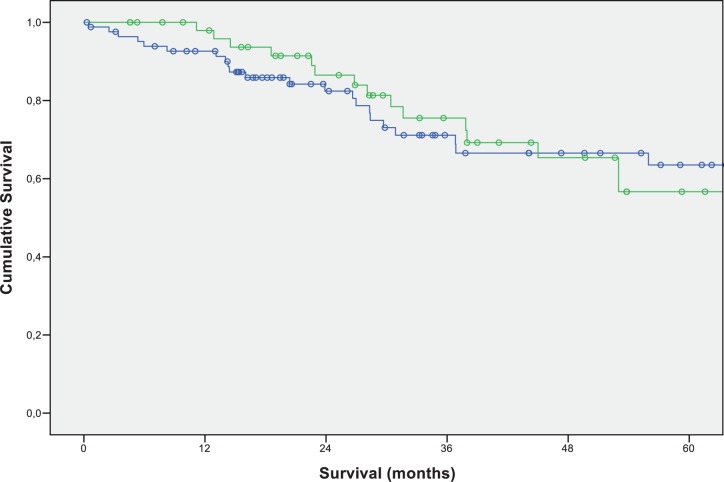
Overall survival-outcome of patients after colorectal liver metastectomy according to occurrence of pulmonary metastases. Green: patients with occurrence of pulmonary metastases (N = 53; 3-year-survival: 75.5%; 5-year-survival: 56.7%); blue: patients without occurrence of pulmonary metastases (N = 84; 3-year-survival: 71.1%; 5-year-survival: 63.5%); (p-value: 0.836).

The median overall survival of all patients was 76.78 months (+/-SD 14.21). The 3- and 5-year survival rate was 72.8% and 60.5%, respectively. Unexpectedly, the 5-year survival rate of patients with pulmonary metastases in addition to CRLM did not differ from the survival rate of patients with CRLM, who had not developed pulmonary metastases (5-year survival rate: without pulmonary metastases 56.7%; with pulmonary metastases 63.5%) (Fig 1).

**Fig 2:**
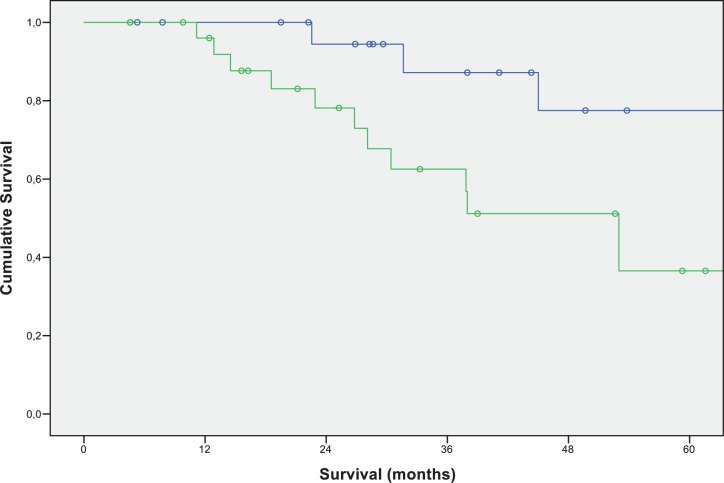
Overall survival of patients after curative liver resection for colorectal liver metastases with additional pulmonary metastases according to the type of treatment. Green: no pulmonary metastectomy (N = 28; 3-year-survival: 62.5%; 5-year-survival: 36.5%); Blue: pulmonary metastectomy (N = 22; 3-year-survival: 87.2%; 5-year-survival: 77.5%) (p-value: 0.015).

When focusing on the group with pulmonary metastases, curative resection of pulmonary metastases resulted in a significant survival benefit. Patients undergoing surgery showed a significantly better 3-year-survival of 87.2% and a 5-year survival of 77.5% compared to 62.5% 3-year survival and 36.5% 5-year survival in patients, who did not undergo pulmonary metastases resection (p-value: 0.015) (Fig 2).

When comparing the survival of patients with pulmonary metastases resection to those not undergoing resection with regard to the primary tumor location (colon / rectum), a survival benefit for resected patients was seen regardless of the primary tumor location. This result did not reach statistic significant values due to too few patients in each group (Colon: p = 0.078; Rectum: p = 0.22).

**Fig 3:**
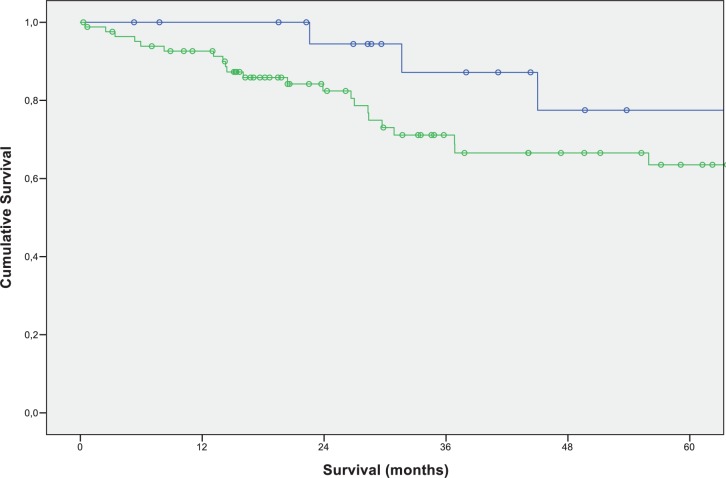
Overall survival of patients after curative liver resection for colorectal liver metastases without development of pulmonary metastases or pulmonary metastases and additional pulmonary metastasectomy. Green: no pulmonary metastases (N = 84; 3-year-survival: 71.1%; 5-year-survival: 63.5%); Blue: pulmonary metastasectomy (N = 22; 3-year-survival: 87.2%; 5-year-survival: 77.5%) (p-value: 0211).

Surprisingly, we found an improved 3- and 5-year survival in patients with resected pulmonary metastases compared to those patients, who did not develop pulmonary metastases at all (3-year survival rate 87.2% vs. 71.1% 5-year survival rate 77.5% vs. 63.5%; p = 0.211) (Fig 3).

**Fig 4:**
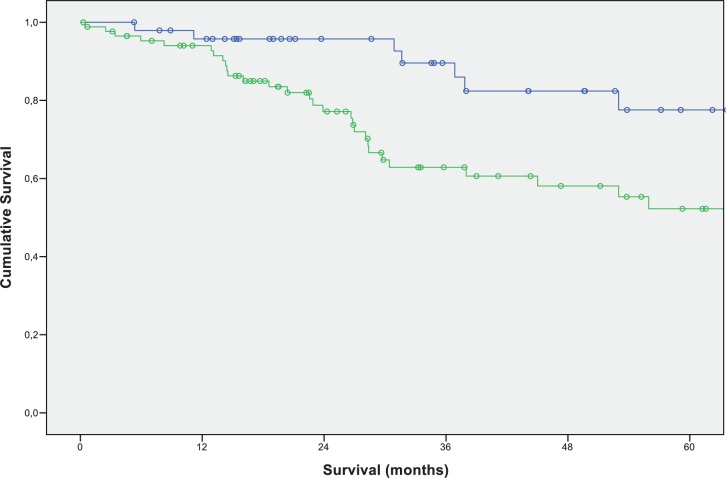
Overall survival of patients with positive N status versus negative N status of the primary CRC after resection of liver metastases (N positive: green (N = 87); N negative: blue (N = 49)).

While we found the N-stages of the primary tumor to be a significant factor for long term survival after resection of liver metastases in multivariate testing, we were unable to identify a predicting factor for the prognosis of patients with pulmonary and liver metastases. In a multivariate analysis of the potential outcome-related factors (CEA-level, N-stage, primary tumor location, time span to occurrence of pulmonary metastases, age), we did not find any statistically significant correlation to an inferior or superior outcome after pulmonary metastasectomy (Fig 4).

## Discussion

During the last decade, the therapeutic options for patients with metastatic colorectal cancer have improved dramatically. New chemotherapeutic agents and improvement in surgical techniques for liver and / or lung metastases resections allow long term survival rates of up to 40% in UICC stage IV patients [15,16].

The surgical options for the resection of liver metastases have improved drastically over the last decade. The resection of single or few metastases has evolved to anatomic major hepatectomies and more recently to extended liver resections, requiring multiple operative steps together with interim induction of hypertrophy of the future liver remnant; i.e. conventional two stage hepatic resections and the ALPPS procedure (Associating Liver Partition and Portal vein Ligation for Staged hepatectomy) [17]. Due to the increased survival in resected patients and potential cure in about 30% of patients with stage IV disease, liver metastasectomy has become the gold standard for treatment of resectable liver metastases, even in bilobar multifocal metastases [11,18–21]. This development resulted in a steadily growing number of stage IV patients, who are considered for hepatic resection.

With increasing numbers of patients considered for and ultimately undergoing surgical resection of liver metastases, as part of multimodal therapy concept for stage IV colorectal cancer, the cohort of patients with a combination of hepatic and pulmonary metastases will increase as well.

Resection of pulmonary metastasized colorectal cancer has clearly been demonstrated to improve survival. However, surgical treatment options for patients with both liver and lung metastases has been discussed controversially in the past [22]. Metastases in more than one distant site has been regarded as a sign of aggressive tumor biology with poor outcome and little chance for long-term survival following surgical treatment. In contrast, others report favorable outcome data for patients undergoing both liver and lung metastasectomy [23]. A recent pooled analysis identified 146 patients in five studies published between 1983 and 2009, who underwent pulmonary metastasectomy after previous liver resection. The five-year overall survival was 54.4%, which was found to be superior to the expected survival of patients with UICC stage IV CRC [7]. This survival is comparable to the observed overall survival in our study of 77.5%.

In the literature previous liver resection has been considered as a negative predictive marker for the oncological outcome following lung metastasectomy [14]. In fact, many of these studies include only a series of consecutive patients and / or were carried out before the introduction of modern chemotherapeutic and biological agents, which might be one explanation for the difference in outcome.

To estimate the prognosis of patients presenting with pulmonary and liver metastases in our own patient population, we retrospectively analyzed all patients, who underwent resection of liver metastases from colorectal cancer at our institution with a special focus on the occurrence and treatment of additional pulmonary metastases. Our results clearly demonstrate that patients with additional pulmonary metastases, who did not undergo resection, experienced an inferior outcome. The overall 5-year survival rate in this group was less than 40%, but nearly 80% for patients who underwent curative resection of their pulmonary metastases. This might be the result of different biological types of tumors, as nearly all patients, who did not undergo pulmonary resection, displayed a diffuse metastatic pattern.

Interestingly, our group of patients showed a better 5-year survival rate compared to the data found in the literature [13,14]. This can be explained by several reasons. First, many patients in our study were also treated with modern multimodal chemotherapy agents, differing from previous studies in the literature [14]. Second, there might be a selection bias in the patients undergoing pulmonary / liver resection. However, based on the registry data, we could not identify any factors varying between the two patient cohorts. Third, except the enhanced 5-year survival rate, which is higher compared to current published data, the disease-free or relapse-free 5-year survival rate was about 30% (data not shown), comparable to the results found in current publications [13,14]. This indicates an improved survival due to the application of new chemotherapeutics and repeat-liver resection, which prolongs the overall survival, but did not influence the recurrence-free survival.

One limitation of this study is the sole comparison of patients undergoing pulmonary resection to patients, who were diagnosed with pulmonary metastases based on growing lesions or newly identified lesions in a CT scan. In retrospect, we were only partially able to evaluate why some patients did not undergo resection despite no significant differences in the number of pulmonary metastases and other demographic factors between both groups. But patients in the non-resected group mainly presented with advanced cancer spread at the point in time of pulmonary metastasis detection, reflecting a worse tumor biology.

Another limitation is that the patients were treated with different chemotherapy protocols and agents, making it impossible to evaluate the chemotherapy impact due to the small study cohort.

Several factors have been proposed to correlate with the survival after resection of pulmonary metastasis, such as the number of metastases CEA levels or the N-stage of the primary tumor [24,25]. For liver metastases, the so-called Fong score and other scoring systems predict survival after resection. One major prognostic factor of the Fong score is the occurrence of lymph-node metastasis combined with the primary tumor. This turned out to be reproducible for liver metastases in our study. However, we did not find an influence of the primary N-stage on the development of additional pulmonary metastases in our patients.

A recent short Meta-analysis by Lamuchi and colleagues including 1669 patients identified elevated preoperative serum levels of carcinoembryonic antigen (CEA), the presence of multiple or bilateral pulmonary metastasis, mediastinal lymph node involvement, and a shorter disease free survival as worse prognostic factors. Unfortunately, we could not reproduce this data in our cohort due to different reasons. Only for a minority of patients the CEA levels were available prior to pulmonary resection. Furthermore, the number of patients in each group was too small to reach valid data [26].

Interestingly, there is also a tendency for patients with both lung and liver metastasis, who underwent successful resection of their metastases, to have a better outcome than patients not developing pulmonary metastases at all. Comparable results have been published by Brouquet in 2011 and Riquet in 2010 [8,27]. One explanation might be an altogether favorable tumor biology leading to the development of single or resectable multiple pulmonary metastases. Furthermore, while pulmonary metastases can be resected and, in principle, cured, most patients with peritoneal carcinomatosis, diffuse lymphatic metastasis or bone metastasis cannot be treated by surgical resection. In line with these results, patients with metastases outside the liver or lung have a worse outcome. Similarly, a large series of patients with pulmonary resection of colorectal metastases showed the occurrence of extra-thoracic metastases as an independent prognostic factor for poor survival [7]. This observation could be the reason why the development of pulmonary metastases *per se* is not associated with a worse outcome.

Furthermore, the resection of pulmonary metastasis leads to a “tumor free timespan” and thereby could reduce the number of CTX and cumulative dose toxicity and could save the opportunity for multimodal CTX in diffuse metastatic stage. In our population, only 6 out of 22 patients (27.3%) showed long term disease free survival (data not shown).

In conclusion, we could show that resection of both pulmonary and liver colorectal metastases led to an excellent long-term survival and should be considered whenever possible. Furthermore, the development of additional resectable pulmonary metastases is not necessarily a poor prognostic marker. In case of synchronous metastases to the liver and lung we prefer a “liver first” approach, due to two reasons. A) to avoid compromised ventilation after abdominal laparotomy, which is the case when pulmonary metastases are resected in advance. B) lung metastases are often small and relative growth during the time delay due to liver resection does not render them inoperable, whereas vice versa the growth of CRLM could lead to an inoperable state. This is especially the case in bipulmonary metastasis where a two stage procedure is intent which will take a timeframe of up to 12 weeks. This data from a retrospective, single institution analysis should encourage multi-disciplinary tumor boards to consider patients with metachronous and synchronous hepatic and pulmonary metastases for surgical resection.
